# Social Isolation and COVID-19 Mitigation: Perspectives of Key Informants in the United States and Japan

**DOI:** 10.1093/geroni/igab046.1705

**Published:** 2021-12-17

**Authors:** Emily Ihara, Megumi Inoue, Cortney Hughes Rinker, Naoru Koizumi

**Affiliations:** 1 George Mason University, Fairfax, Virginia, United States; 2 George Mason University, Arlington, Virginia, United States

## Abstract

The deleterious health effects of social isolation and loneliness among older adults have been well-established and were exacerbated by the forced separation for those at health risk of contracting the COVID-19 virus. Both the United States and Japan are experiencing phenomenal growth of the older adult population; Japan is considered a “super-aged” society, with the highest proportion of people aged 65 and older in the world. This study examined how COVID-19 and mitigation measures may have affected services for older adults. We conducted key informant interviews with specialists in aging and older adult care in both Japan (n=5) and the United States (n=14). All interviews were conducted over Zoom and lasted 30-60 minutes. The research team transcribed and checked the interviews for accuracy and conducted multiple coding sessions to identify, sort, and consolidate the codes using Atlas.ti. Key themes in both countries that emerged included the many cracks in the system of programs and services for older adults, the inaccessibility to technology and the internet, and the particular difficulties of socioeconomic inequities, especially for those living alone. Older adults were motivated to become more technologically proficient and local communities came forward to help provide support. One key informant from the U.S. noted that their organization experienced a 600% increase in interest among volunteers as a result of the pandemic. Despite the many challenges of the pandemic, many silver linings emerged. One participant poetically stated, “I think that's human nature – when you have no other choice, you find a way.”

